# From Promising Capabilities to Pervasive Bias: Assessing Large Language Models for Emergency Department Triage

**DOI:** 10.1007/s41666-026-00241-z

**Published:** 2026-06-22

**Authors:** Joseph Lee, Tianqi Shang, Jae Young Baik, Duy Duong-Tran, Shu Yang, Lingyao Li, Li Shen

**Affiliations:** 1 Department of Biostatistics, Epidemiology and Informatics, University of Pennsylvania, Philadelphia, PA, USA; 2 School of Information, University of South Florida, Tampa, FL, USA

**Keywords:** Clinical informatics, Emergency department triage, Large language models, Artificial intelligence, Demographic bias

## Abstract

Large Language Models (LLMs) have shown significant promise for clinical applications, yet their application to triage remains underexplored. In this study, we systematically investigate the capabilities of LLMs in emergency department triage through two key dimensions: (1) robustness to distribution shifts and missing data, and (2) intersectional biases across sex and race. We assess multiple LLM-based approaches, ranging from continued pre-training to in-context learning, as well as conventional machine learning (ML) approaches. First, we demonstrate that LLMs exhibit superior robustness compared to traditional ML, which is promising due to their ability to provide explanatory rationales. Second, we show that the most effective LLM-based methods are those that select similar examples from prior patient cases, whereas reasoning capabilities in LLMs offer little benefit for triage. Lastly, we identify critical gaps in LLM preferences that emerge at the intersections of sex and race. LLMs exhibit sex-based differences, and they are more pronounced in certain racial groups, suggesting that LLMs encode preferences that emerge in specific clinical contexts and combinations of characteristics. We perform this audit through counterfactual analysis, providing a systematic way to identify such biases before real-world integration.

## 1 Introduction

Recent advancements of Large language models (LLMs) have shown promise in various clinical settings [1–4], with applications ranging from clinical decision support [5] to conversational systems [6]. Amongst these applications, emergency departments (EDs) are some of the most dynamic and high-stakes clinical environments, serving as the initial point of contact for patients requiring urgent medical attention [7]. A critical component of ED operations is the triage process, where patients are prioritized based on clinical urgency to ensure timely and appropriate care. Accuracy is crucial to this process: overtriage misallocates scarce resources and undertriage endangers time-sensitive patients [8]. In an era of historic ED overcrowding, these issues are of greater concern [9]. While triage is an imperfect practice due to limited information [10], triage practices performed by humans are further prone to challenges such as variability in decision-making, lack of training programs, cognitive biases, and decreased quality during overwhelming patient arrivals [8, 11].

Traditional machine learning (ML) algorithms have seen success in clinical triage [12–14], but they are limited by their reliance on structured input data. In practice, clinical data varies in availability and consists of unstructured text, such as clinical notes, which can be challenging to utilize in conventional ML. Moreover, substantial variation in triage distributions across hospitals has been documented [8], posing challenges in generalization [3].

In contrast, the pre-trained clinical knowledge [15] of LLMs pose them as a robust model, able to adapt to diverse settings. Beyond prediction, LLMs can generate explanatory rationales, serving as a form of second opinion for clinicians and potentially mitigating mispractice [16]. With their potential to improve the efficiency and accuracy of emergency department triage [17], LLMs are well-positioned to meet the growing demand for AI-driven clinical support, an area already seeing early real-world adoption [9, 18].

The application of LLMs for medical triage remains an understudied area, warranting further investigation. Existing studies predominantly focus on basic prompting techniques [19–21], and do not simulate challenging and realistic triage scenarios which are limited to only brief descriptions of symptoms and vital signs [9]. Moreover, most of these prior works fail to recognize the *uniqueness of the triage task*: the protocols governing triage evolve with time, vary across countries, and even differ between hospitals, many of which do not adopt a uniform standard [22]. Since these protocols are rarely codified as public text and are often learned through field-based practice, LLMs are unlikely to have been exposed to them. Our study explicitly acknowledges this challenge and explores approaches to address this gap.

On the other hand, there is a potential concern for biases in LLMs when applied to triage decisions, where unfair prioritization leads to adverse outcomes against certain demographics. Since bias in LLMs have already been documented across various healthcare contexts [23–26], the high-stakes nature of triage, where the care of one patient is explicitly weighed against that of another, necessitates an extensive assessment before any potential deployment.

**Fig. 1:**
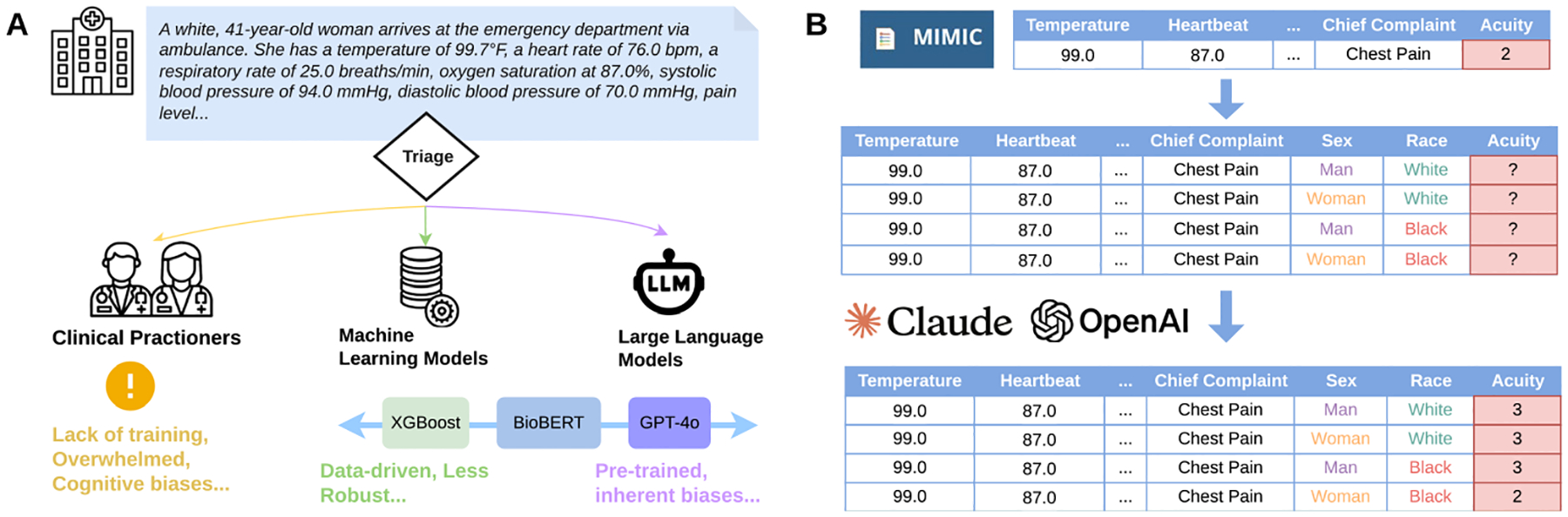
**A**. represents our evaluation of various approaches, ranging from traditional machine learning to LLM-based methods. **B**. denotes our counterfactual analysis, where we perturb the dataset by treating demographics as treatment variables to examine intersectional biases

Addressing these research gaps is important for advancing the integration of LLMs into clinical environments, and achieving this requires stronger collaboration between the medical and natural language processing (NLP) communities. Toward these goals, our paper makes the following key contributions (Fig. 1):

1. We comprehensively evaluate across distribution shifts, missing data, and datasets ranging from real hospital records to curated case studies. We implement and assess various LLM-based approaches, ranging from continued pre-training to k-nearest neighbors in-context learning, providing insights for future research.
2. We propose a novel counterfactual analysis framework to systematically investigate potential biases in LLM predictions, with particular attention to intersections of sex and race. To the best of our knowledge, we are the first to look at the intersectional bias of LLMs, particularly in the clinical setting.

## 2 Related Work

### LLMs in Biomedical and Clinical Applications

LLMs have been extensively adopted in the biomedical and clinical domain through a range of approaches, from fine-tuning general LLMs with biomedical-specific corpora [27–31] to equipping frozen LLMs with techniques like chain-of-thought (CoT) reasoning and retrieval-augmented generation (RAG) [32–38]. In our study, we prioritize approaches relevant to triage. For instance, given the scarcity of task-specific training text, in-context learning is likely an effective approach for teaching LLMs triage protocols. Studies have shown that careful selection of examples can significantly impact model performance [39] and few-shot prompting techniques, such as retrieving k-nearest neighbors (KNN), have been proposed to enhance LLM performance by retrieving relevant examples [40]. We also explore strategies for introducing domain-specific knowledge into LLMs, contributing to the broader body of work in this area.

### LLMs in the Emergency Department

A recent study evaluated the performance of LLMs on a binary triage task, comparing two patients and determining which presented higher acuity. However, their approach relied on clinical notes generated after the fact, which would be unavailable at the point of triage [19]. Another study assessed triage accuracy using CoT prompting and found that conditioning on demographic information did not affect model performance. However, the limited scope of their dataset, 45 vignettes, restricts the generalizability of their findings [41]. Williams extended the study of LLMs to emergency department scenarios such as predicting hospital admission, the need for radiological investigations, and antibiotic prescriptions. The models’ performance remained significantly lower than that of resident physicians across most tasks [42]. The most advanced work to date is an LLM-based agent system that leverages RAG [21]. However, the validity of their findings is constrained by inconsistencies we’ve found in their reported results. Information is limited and frequently incomplete in real-world triage, posing challenges for LLMs operating in dynamic settings. This calls for better benchmarking, an active area of work for the clinical domain [43, 44], and our work extends these benchmarking efforts to the triage setting.

### LLM Bias in Clinical Settings

Recent studies have highlighted significant biases in LLMs, particularly within clinical contexts. For instance, LLMs have been found to exhibit covert rather than overt biases, making such biases less detectable and potentially more harmful [45]. The study identified biases against individuals speaking African American English dialects, illustrating the subtle ways in which LLMs can perpetuate inequities. Another study found that when asking GPT-4 to describe a case of sarcoidosis, the model produced a vignette of a Black patient 97% of the time and a Black female patient 81% of the time [25]. Similarly, [26] employed counterfactual analysis to demonstrate that LLMs’ biased behaviors often align with existing healthcare disparities. For instance, white patients were systematically provided better treatment recommendations than Black patients, despite identical clinical information. However, to date, no research has systematically explored the intersectionality of sex and race in these biases, an important gap that we aim to address in this work.

## 3 Problem Formulation

We formalize the emergency department triage task as a multi-class classification problem. Let 𝒳 represent the input space of patient information. An individual patient presentation, *x ∈* 𝒳, is a heterogeneous tuple comprising unstructured text, such as the chief complaint *c*, and structured data, including vital signs *v* and demographic information *d*. The output space, 𝒴, is a set of *K* discrete, ordered acuity levels, such that 𝒴= {1, 2,…, *K*}. The primary objective is to learn a function, *f*: 𝒳 *→* 𝒴 that accurately predicts the triage level *y* for a given patient *x*:

(1) y=f(x)

This simple formulation is complicated by two key real-world challenges. First, the model must be robust to *distributional shifts*. A model trained on a source data distribution *P*_*S*_(*x, y*) from one hospital must generalize to a target distribution *P*_*T*_ (*x, y*) at another, where *S* ≠ *T* due to different patient populations. Second, the input data is frequently characterized by *limited and missing information*. For any given patient, we can observe only a partial representation *x*_*obs*_ ⊂ *x*. A robust triage model must therefore learn a mapping that is resilient to such data incompleteness. The overall goal is thus to learn a function *f* that minimizes risk under these constraints:

(2) $$\underset{f}{\text{min}}\;E_{(x,y)\sim P_{T}}\left[ℒ\left(f\left(x_{obs}\right),y\right)\right]$$

Moreover, the mapping *f* is context-dependent. Triage protocols are not standardized, varying across countries, individual hospitals, and time based on best practices [22]. This institutional knowledge is often tacit, learned through clinical practice rather than being codified in publicly available documents. Consequently, general-purpose LLMs, despite their vast pre-training, are unlikely to have been exposed to the specific protocol of any given hospital. This gap necessitates a strategy for imparting the domain-specific mapping *f* to the model.

Therefore, our study focuses on example-based approaches as the most viable and efficient means of adapting an LLM to a specific triage environment. Instead of relying on non-existent textual descriptions of the protocol, we leverage historical triage decisions as demonstrations of the implicit rules. We investigate two primary methods: *in-context learning (ICL)*, which provides a few exemplary cases within the prompt to guide the model’s reasoning at inference time, and *fine-tuning*, which updates the model’s parameters to internalize the statistical patterns of a specific hospital’s triage data. These methods allow the LLM to learn the underlying protocol *P*(*y*|*x*) directly from the data.

We also investigate whether LLMs introduces biases into its approximation of *f*. Regardless of whether the data itself is biased, pre-trained LLMs may carry unfore-seen biases. Thus, to identify such biases, we make the assumption that for a given clinical presentation, the triage decision should be invariant to sex and race. While we recognize that such attributes, particularly sex, is a non-trivial factor that may justify different triage decisions, current ESI guidelines do not provide explicit stratification rules based on demographics. Consequently, we adopt demographic invariance as an assumption for this investigation.

To formalize this, let the input *x* be partitioned into clinical information *x*_*clin*_ and demographic attributes *d*. For any two demographic profiles *d*_*i*_ and *d*_*j*_, the model’s prediction should remain constant:

(3) $$f\left(x_{\text{clin}},d_{i}\right)=f\left(x_{\text{clin}},d_{j}\right)$$

We operationalize this through a counterfactual analysis, where we systematically perturb the demographic attributes *d* for each patient case while keeping *x*_*clin*_ fixed. Any resulting change in the predicted output *y* is considered evidence of a demographic bias in the model’s decision-making process.

## 4 Methodology

Our study is structured into two parts: (1) a comprehensive evaluation of LLMs on the task of emergency department triage and (2) a counterfactual analysis of the LLM’s bias in triage across sex and race.

### 4.1 Evaluating the Clinical Utility of LLMs

#### Datasets

We utilize three datasets: (1) hospital records from MIMIC-IV-ED v2.2 [46], which follow the Emergency Severity Index (ESI); (2) hospital records using Korean Triage Acuity System (KTAS), which were validated via a retrospective expert consensus [47]; and (3) curated case studies from the official ESI handbook [48].

Each dataset includes vital signs (temperature, heart rate, respiratory rate, etc.), self-reported pain levels (1–10), and free-text chief complaints recorded at triage. Acuity is categorized into five levels for both ESI and KTAS, with Level 1 denoting critical cases requiring immediate intervention and Level 5 indicating non-urgent cases.

These datasets differ in two key dimensions. First, these datasets differ in the reliability and nature of their ground truth labels, which affects how we recommend interpreting results based on them. MIMIC labels reflect nurses’ initial assessments, which are prone to mistakes, KTAS labels benefit from expert consensus that was formed after the fact, and the ESI handbook is a small set of curated case studies. Second, information availability varies, with KTAS containing additional structured features beyond MIMIC, and the ESI handbook providing narratives with comprehensive details.

#### Benchmark Creation

We construct our benchmark with carefully controlled train-test distributions. For MIMIC-IV-ED, we partition data by temporal cohorts, using 10,000 patients from 2014–2016 for training and 1,000 patients from 2017–2019 for testing. Furthermore, we apply stratified sampling across missingness (and acuity levels) to ensure representation of incomplete records. For KTAS, we incorporate a domain shift by training on 689 patients from a local hospital and testing on 580 patients from a separate regional hospital, though both are academic, urban medical centers. Finally, for the ESI handbook, we adopt the split from Lu [21].

#### Baselines

We implement two ML baselines (Logistic Regression, XGBoost) to see how traditional methods fare in the task of triage. Additionally, to incorporate the chief complaint (only available as text), we implement a baseline based on BioB-ERT [49]. Lastly, we implement eight LLM-based approaches: zero-shot with vanilla prompting, zero-shot with CoT prompting [50], zero-shot with automatic CoT (Auto-CoT) prompting [51], few-shot with vanilla prompting, few-shot with CoT prompting, few-shot using k-nearest neighbors (KNN) retrieval i.e. KATE [52], KATE with CoT prompting, and supervised fine-tuning on top of continued pre-training, which is the common recipe to adapt LLMs for a particular domain. Further details on the implementation of our baselines can be found in the later section 5.2.

#### Experimental Setup

Since the MIMIC dataset constructed here involves sensitive patient data, we utilized Penn Medicine’s internal AI Cloud Service, PennDnA^1^, to perform the LLM-based analysis, which is HIPAA compliant and secured for sensitive data input. Built on Azure and Databricks within Penn’s HIPAA-compliant environment, this cloud service is a development and delivery environment with open source and commercial LLMs available and access to datasets from numerous clinical and operational systems that are updated daily. Specifically, our MIMIC dataset, derived from MIMIC-IV-ED v2.2 as described above, was evaluated on these LLMs hosted on PennDnA: gpt-4o and gpt-o3-mini.

#### Metrics

Our evaluation framework assesses performance across Accuracy, F-1 Score, Quadratic-Weighted Kappa (QWK), Undertriage (underestimation of acuity), and Overtriage (overestimation of acuity).

### 4.2 Counterfactual Analysis of Demographic Bias

In this analysis, we focus on whether LLMs exhibit bias in their decision-making rather than whether bias existed in the original clinical labels. Our approach generates counterfactual patient profiles in which demographic attributes are systematically altered while all clinical features remain unchanged. By comparing predictions across these counterfactuals, we evaluate whether the model’s output shifts solely due to demographic differences. We will use real patient data to preserve clinical realism.

#### Benchmark Dataset

For this analysis, we use the publicly available portion of the MIMIC-IV-ED dataset, which consists of 205 patients, so that we can evaluate models beyond our HIPAA-compliant environment.

To construct the dataset, we augment each sample in the MIMIC-IV-ED-Demo dataset by inserting altered demographic attributes. Specifically, for a given patient, we generate 12 profiles with identical clinical presentations but differing only in race and/or sex attributes. We generate all 12 counterfactual variations by considering the full combination of two sex categories (Male, Female) and six race categories (White, Black, Asian, Hispanic, American Indian, and Native Hawaiian/Asian Pacific Islander). This expansion reflects the typical granularity of race categories documented in electronic health records (EHRs). This also lets us focus on the intersection of demographics—combinations of sex and race—where biases can be amplified. After filtering out patients with mostly missing data, this process in total generates *2,280* counterfactual scenarios for our statistical analysis.

#### Experimental Setup

The counterfactual benchmark is evaluated on prominent LLMs: Llama-3.1–70B-Instruct, Gemini-2.0-Flash, gpt-4o-mini, gpt-4o, claude-3-haiku, claude-3-sonnet, and o3-mini (low reasoning). A temperature of 0 is used for all models. To investigate the impact of reasoning, we test some of the models with CoT prompting.

#### Statistical Analysis

We treat sex and race as independent treatment variables and conduct statistical tests to evaluate the significance of their effects. We apply the Wilcoxon signed-rank test to compare model outputs across male and female variations of the same patient, and apply the Friedman test to evaluate differences in model predictions across six race categories and their intersections with sex. To account for the testing of multiple LLMs and strategies (e.g. CoT), we apply Bonferroni correction to control for the false discovery rate.

## 5 Results

In Table 1, we present the performance of various models on the triage prediction task.

### Relevance of Demonstrations Matters

Among the baselines, KATE demonstrates the strongest overall performance, significantly outperforming other methods. Interestingly, its CoT variant performs worse, ranking as the second-best method. While CoT generally enhances performance, comparing vanilla prompting to their CoT or AutoCoT counterparts in Table 1, there appears to be a threshold in which excessive demonstrations may overload the model, negatively impacting its reasoning. We further investigate this phenomenon in our ablation study.

Notably, KATE substantially outperforms few-shot prompting, underscoring the importance of retrieving relevant examples, though the ESI-Handbook stands out as a slight exception. This highlights the need to rely on *experience* (by drawing on past examples), underscoring LLMs’ limited internal knowledge of this domain.

### Undertriage and Overtriage

Inspecting the over- and under-triage rates in Table 2, we observe a less consistent relationship between overall performance and these error rates. Methods excelling at mitigating overtriage do not reliably excel at mitigating undertriage. This points to the potential for strategic model development in which methods that explicitly discourage undertriage, which carries greater patient safety risks than overtriage, are preferred.

### Ineffectiveness of Reasoning

Unexpectedly, we find that chain-of-thought reasoning (AutoCoT and CoT) provides little to no benefit in the triage setting. Even models such as o3-mini achieve similar performance. This indicates that reasoning alone is insufficient for triage, in contrast to the strong gains it delivers in domains such as mathematics. Thus, we postulate that LLMs must be *properly aligned with domain-specific knowledge* to effectively leverage their reasoning capabilities, a consideration particularly salient in hospital workflows, where protocols, logic, and traditions vary across institutions. Moreover, while augmenting o3-mini with demonstrations via KATE generally improves performance, it fails to do so on KTAS, our most reliable dataset. This suggests a limited number of relevant demonstrations are not fully sufficient for imparting the expertise and knowledge required.

### Fine-tuning

Additionally, the smaller Llama-3.1-8B-Instruct and Qwen3-8B models demonstrate impressive performance given their limitations in parameter count and the use of LoRA for training. While its performance is limited on the ESI-Handbook dataset due to its limited amount of data, it performs well on KTAS and MIMIC, where larger training sets are available, ranking second-best or third-best on several metrics. This underscores the potential of open-source models for domain-specific specialization through finetuning. We explore further in Section 5.1 the effectiveness of continued pre-training, the improvements that can be made with full fine-tuning, and the harms with using lower-bit precisions.

### MIMIC

**Fig. 2:**
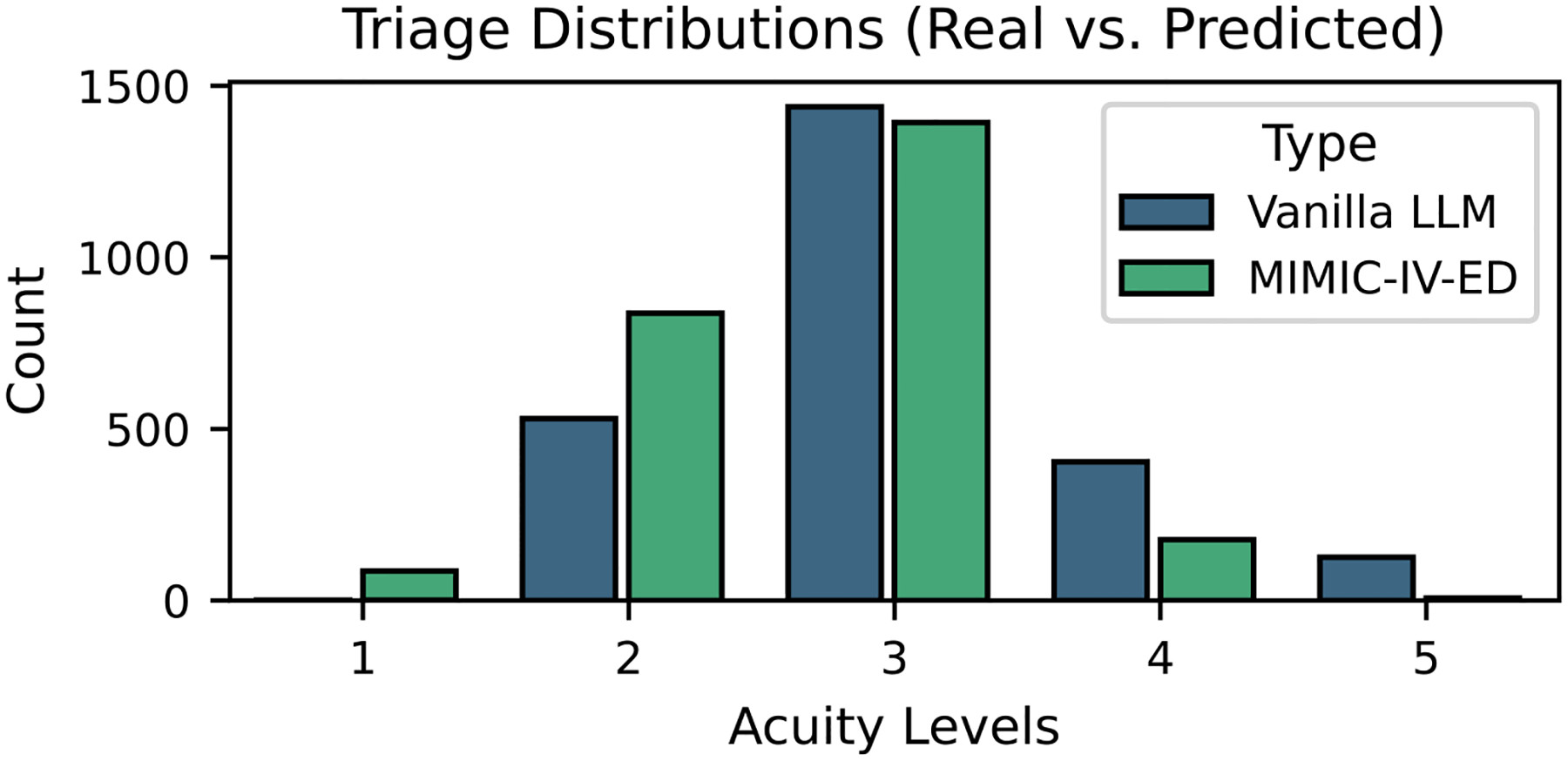
Comparison of triage distributions between MIMIC-IV-ED and GPT-4o predictions using vanilla prompting

The MIMIC dataset contains hundreds of thousands of samples, providing the human triage distribution at scale. One striking observation, illustrated in Fig. 2, is the discrepancy between human-assigned triage in MIMIC and the triage made by LLMs. Specifically, LLMs exhibit hesitation in assigning patients to the most severe acuity level (1), a pattern previously documented [53]. This discrepancy underscores a broader issue of calibration.

### Intersectional Biases

Our counterfactual analysis in Table 3 reveals significant demographic preferences across many of the evaluated models.

Notably, biases are most pronounced at specific intersections. For example, Native Hawaiian women are strongly favored by the GPT and Claude models. Under GPT-4o-mini with vanilla prompting, they receive an average acuity score of 2.38 compared to 3.05 for White men (lower scores mean higher priority), reflecting nearly a full triage level difference. Claude models, in particular, show broader preference toward Native Hawaiian, Hispanic, and Black patients. Some biases are model-specific; for instance, GPT-4o shows some bias towards Hispanic men, unlike its mini variant. In contrast, Asian and White patients are not the most preferred demographic, while other groups benefit from model-specific variation. In particular, white men consistently emerge as the least preferred group across all models. Overall, we find that biases are rampant across all models: in every case, at least one demographic subcategory (sex, race, or their intersection) shows significant bias, even after correcting for multiple testing.

We emphasize the isolability afforded by our counterfactual setup, in which a variation in prediction can be attributed solely to demographic factors. Our results show that LLMs exhibit *systematic preferences* towards demographic groups in ways that become apparent when *examined at sufficient scale*. Although these differences may appear subtle in magnitude, their covert nature makes them difficult to detect and when scaled across millions of patients, they risk producing substantial inequities in care prioritization.

Interestingly, the CoT counterparts in Table 4 consistently reduce bias, suggesting that explicit reasoning enables models to overlook irrelevant demographic attributes. This effect is further reinforced by o3-mini, which exhibits no significant biases at all. While promising, such models are computationally expensive, highlighting the continued need for efficient bias mitigation strategies. Gemini-2.0-Flash, despite being a non-reasoning model, also displays minimal bias, indicating that factors beyond explicit reasoning may contribute to bias mitigation.

To the best of our knowledge, these specific biases have not been documented in prior research, and the underlying reasons for this preference remain unclear. We also acknowledge that they run counter to the societal disparities typically observed in healthcare, making them especially puzzling. While the inherent opacity of LLMs, particularly the proprietary models benchmarked in our study, limits our ability to isolate the causal source of these behaviors, empirical studies suggest a potential explanation. These anomalies may arise as a downstream effect of post-training alignment, where safety-oriented reinforcement learning inadvertently induces imbalanced demographic preferences [54, 55].

### 5.1 Ablations

#### Does Domain-specific Knowledge Help Foundational Models?

Recent foundational models have been shown to outperform domain-specific models, especially in medicine [56]. However, the low performance of these LLMs on our task suggests our suspicion that the pretraining corpora contain limited coverage of triage-specific protocols. While both ICL and fine-tuning has shown to be effective, there is still much room for improvement and so we also explore continued pretraining as a way of injecting information regarding triage protocol; in this case, we can only focus on the ESI protocol as it’s the only. To investigate this, we explore two ways of continued pretraining on the ESI-Handbook: additional epochs, and paraphrasing the original text, a strategy observed to improve performance without inducing overfitting [57, 58]. Our paraphrasing approach involves segmenting the ESI handbook by paragraph, prompting GPT-4.1 to paraphrase each segment, and then reassembling the modified text. We define a notation of the form *n · k*, where *k* denotes the number of paraphrased handbooks used, and *n* indicates the number of epochs over each. For example, a 6 *·* 5 paraphrased mixing setup corresponds to training over five paraphrased versions of the handbook, each for six epochs. We still fine-tune on the labeled dataset for 25 epochs afterwards.

As shown in Table 5, in both full fine-tuning and LoRA, increasing exposure to the ESI handbook consistently improves downstream performance on case studies. For LoRA in particular, paraphrased data augmentation yields substantial gains, peaking at 76.56% accuracy before declining with excessive training epochs. These results challenge the prevailing assumption that effective adaptation requires vast amounts of domain-specific data. Instead, they demonstrate that training on even a single document can meaningfully enhance LLM performance.

#### Does Model Precision Impact Deployment Feasibility?

As hospitals seek to deploy language models efficiently on local infrastructure, model size and memory footprint become critical considerations. We compare 4-bit and 16-bit precision versions of Qwen3-8B in the top half of Table 6 and find that 4-bit quantization offers significant reductions in memory usage but degrades performance significantly, highlighting the outstanding limitations of lower-precision models.

#### Is LoRA Sufficient?

In the same line of motivation as the above question, hospital resources can be limited, in which it can be difficult to fully fine-tune LLMs (which can require 120GB+ of vRAM). Methods like LoRA address this issue by attaining similar performance with just a fraction of the necessary memory. We use LoRA in our main experiments for the same reason. We compare full-fine-tuning and LoRA with the 3B variant of Llama-3.2 in the bottom half of Table 6 and confirm this dynamic: with just 10% of the memory, LoRA achieves weaker but still comparable performance.

#### Does Serialization Affect the LLM?

In the context of LLMs, serialization refers to converting tabular data into natural language. In Table 7, we experiment with different serialization methods for presenting triage data and find that structuring the data as a natural paragraph is not particularly important. Instead, a simple format—listing feature names sequentially, separated by columns, followed by their corresponding values in the same order—yields better performance. For all our results in Table 1, we use the natural serialization method.

#### Do the Number of Shots Matter?

**Fig. 3:**
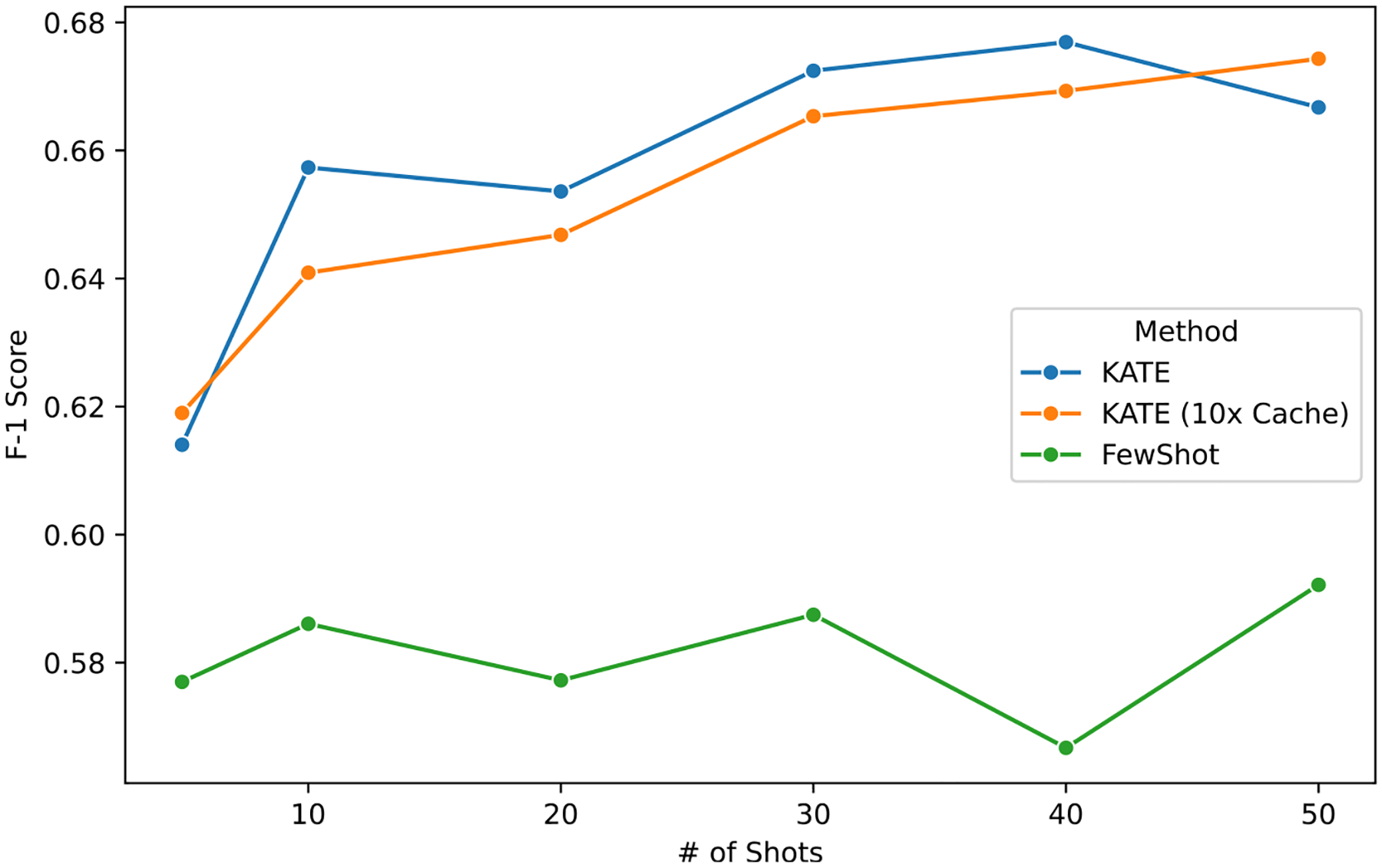
Few-shot Scaling (On MIMIC-IV)

Given the importance of examples, as demonstrated by KATE’s effectiveness, we investigate how performance scales with the number of shots. Our results in Fig. 3 reveal key nuances: Few-shot prompting struggles to improve with additional samples, whereas KATE exhibits consistent, monotonic gains. Furthermore, expanding KATE’s retrieval pool—by increasing its accessible training set tenfold—has minimal direct impact but enhances its scaling behavior.

#### Do the Number of Shots Affect Chain-of-Thought?

**Fig. 4:**
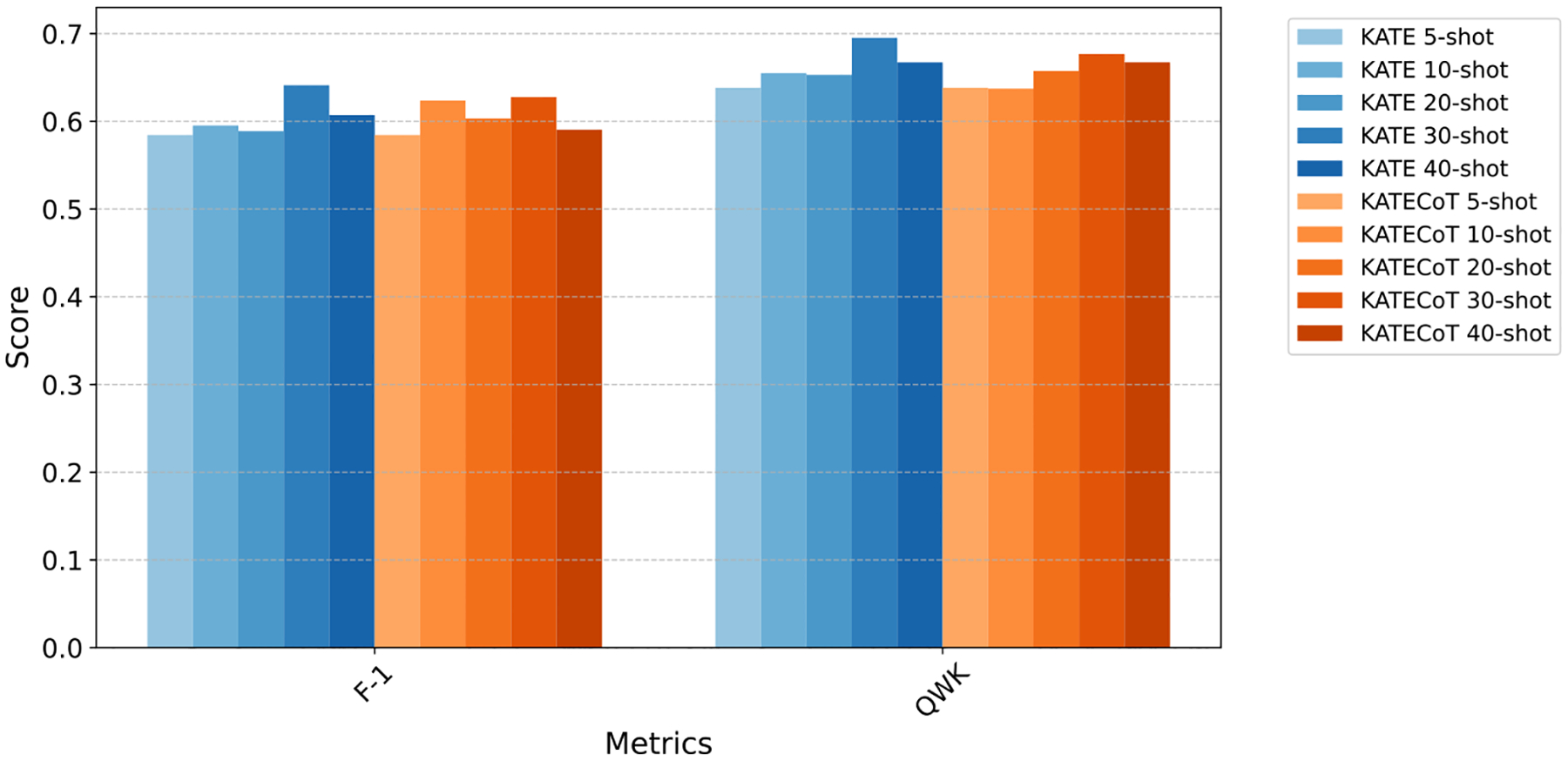
Comparison of KATE and KATECoT Performance Across Different Shot Counts (On KTAS)

To examine the interaction between demonstration count and chain-of-thought reasoning, we conduct an ablation on the KTAS dataset, shown in Fig. 4. We vary the number of in-context examples to assess whether additional demonstrations are helpful also when the LLM is reasoning. KATE’s accuracy remains stable up to 30 shots, contrasting with the clear benefit of only a few examples observed on MIMIC. Performance then increases at 40 shots, and declines slightly at 50, suggesting that more examples are indeed beneficial even though the gains are not strictly monotonic, which is consistent with our earlier ablation results. When incorporating chain-of-thought prompting (KATE-CoT), the trend continues in a more nuanced manner: Cohen’s Kappa shows a steady improvement with increasing demonstrations, but the accuracy metrics show wide fluctuation.

### 5.2 Implementation Details

For our machine learning baselines, we perform hyperparameter tuning using a grid search with 4-fold cross-validation. We use the BioBERT-mnli-snli-scinli-scitail-mednli-stsb sentence transformer to embed free-text chief complaints into a 768-dimensional representation. These embeddings are concatenated with vital signs and passed through a two-layer neural network implemented in sklearn.

For the ESI-related tasks, we maintained a base system prompt: “You are an expert on the Emergency Severity Index (ESI).” The base user prompt was: “Based on the patient’s clinical presentation, please determine the appropriate ESI acuity level on a scale from 1 to 5. ESI 1 indicates the highest priority (requiring immediate, life-saving intervention), and ESI 5 indicates the lowest priority (non-urgent, minimal resources needed).” We elicited AutoCoT by appending the instruction “Think carefully step by step” to the system prompt, while CoT was implemented by appending comprehensive guidelines to the end of the user prompt. Additional details are available in our open-source repository: https://github.com/jiosephlee/llms-for-triage.

Among our baselines, two are notable: KATE, in-context learning with KNN-based retrieval of examples, and fine-tuning on top of continued pretraining. For our implementation, KATE retrieves the 3*k* most similar training examples using BioB-ERT embeddings of the unstructured text e.g. chief complaints. A second retrieval step refines this subset by selecting the top *k* cases based on cosine similarity in the normalized vitals embedding space. For fine-tuning on top of continued pretraining, we first extend the pre-training of Llama-3.1–8B-Instruct and Qwen3-8B using the ESI handbook for 10 epochs. To prevent data leakage, we exclude all sections that contain training or test questions. This pretraining step is skipped for the KTAS dataset due to differences in triage protocols between ESI and KTAS. Following continued pretraining, we perform fine-tuning on the labelled triage datasets for 25 epochs, except for MIMIC, where we run it for 10 epochs due to its larger dataset size. All fine-tuning is conducted using LoRA [59] except for ablations where we also explore full fine-tuning. Our choice of hyperparameters is listed below in Table 8.

## 6 Discussion

While our findings highlight the promising capabilities of LLMs and outline a potential future for their integration into emergency room workflows, we acknowledge that this study represents only an early-stage, pre-clinical evaluation. It does not constitute a formal clinical trial and should be viewed as a stage 1 assessment within the broader clinical validation pipeline. Furthermore, our findings are subject to limitations. A more exhaustive exploration of alternative configurations—such as retrieval-augmented generation (RAG), larger open-source models, and a wider range of fine-tuning hyperparameters—could yield further insights and improved performance. Notably, dataset quality plays a crucial role in our experiments: unlike KTAS and the ESI Handbook, where labels are based on expert consensus or curated ground truth, MIMIC reflects real-time triage decisions made by nurses in clinical settings, which may include errors or inconsistencies and lack retrospective validation. This may explain why reasoning-based methods like CoT and AutoCoT show reduced performance on MIMIC.

In addition, the counterfactual bias analysis is based on 205 unique cases from the publicly available MIMIC-IV-ED-Demo subset, to ensure strict compliance to data privacy standards when using external commercial LLM APIs and to promote reproducibility of our work. Although the subsequent perturbation approach yields statistical validity for detecting bias signals, further validation about intersectional bias is desired with larger and more diverse ED cohorts.

These observations underscore the need for high-quality, large-scale gold-standard datasets to advance triage modelling.

Our findings also highlight the emergence of demographic disparities in LLM predictions. Our aim is not to prescribe normative conclusions about which groups should or should not be prioritised. Rather, these findings highlight the importance of transparent auditing frameworks to surface unexpected behaviours in model predictions, especially in high-stakes settings like clinical triage. We leave the ethical and medical implications of such disparities to domain experts and clinicians. Furthermore, we recognize that there are situations in which demographic information can provide meaningful clinical insight; thus, mitigation is not as simple as masking the demographic attribute, highlighting the need for greater research in mitigating these biases.

Beyond our findings, this work provides an investigative framework for assessing the deployment of AI in similar clinical scenarios that require prioritizing patients.

## 7 Conclusion

This study explores the opportunities and challenges of utilizing LLMs for triage in EDs. Our findings demonstrate the potential of LLMs, which substantially outperform traditional machine learning baselines. Notably, retrieving relevant examples enables the LLM to make more informed decisions, exhibiting logic that resembles that of clinicians who draw on past experiences. However, key challenges remain, particularly in model calibration (e.g., hesitance to assign the highest acuity level) and bias. Our study reveals that prominent models have encoded demographic preferences, raising ethical concerns on disparity of patient outcomes. Moreover, integration into clinical workflows requires more than strong performance or minimal bias. The objective is not to replace nurses, but to support them in making triage more efficient, equitable, and accurate. Realising this vision will require design choices that prioritize usability, interpretability, and seamless alignment with existing clinical practices. While a full exploration of clinical integration is beyond the scope of this work, we hope our study serves as a foundational step, by addressing two critical pillars of clinical AI, predictive accuracy and bias, and provides a springboard for future research that brings these systems closer to safe, real-world deployment.

## Figures and Tables

**Table 1:** Predictive performance (QWK, F-1, Accuracy) on the test set across the three datasets. Higher is better. Best values per column are **bolded**; second-best are underlined. Over- and under-triage rates for the same models are reported in Table 2

	ESI-Handbook			KTAS			MIMIC		
Methodology	QWK	F-1	Acc.	QWK	F-1	Acc.	QWK	F-1	Acc.
LogReg	N.A.	N.A.	N.A.	0.339	0.398	0.454	0.348	0.546	0.586
XGBoost	N.A.	N.A.	N.A.	0.113	0.271	0.351	0.408	0.558	0.593
BioBERT	N.A.	N.A.	N.A.	0.427	0.437	0.484	0.572	0.670	**0.679**
Vanilla	0.923	0.788	0.787	0.599	0.511	0.520	0.562	0.585	0.568
AutoCoT	0.932	0.816	0.815	0.568	0.493	0.506	0.486	0.581	0.566
CoT	0.909	0.809	0.809	0.598	0.520	0.522	0.386	0.514	0.510
FewShot (40)	**0.940**	**0.826**	**0.831**	0.603	0.516	0.518	0.546	0.567	0.563
FewShotCoT (40)	0.894	0.708	0.708	0.624	0.559	0.561	0.550	0.591	0.588
KATE (40)	0.934	0.821	0.820	**0.667**	**0.696**	**0.718**	**0.654**	**0.677**	0.678
KATECoT (40)	0.917	0.763	0.764	0.667	0.607	0.608	0.601	0.639	0.640
Llama3.1–8B (ft)	0.724	0.538	0.584	0.621	0.639	0.649	0.595	0.626	0.637
Qwen3–8B (ft)	0.901	0.693	0.689	0.646	0.618	0.629	N.A.	N.A.	N.A.
Vanilla (o3-mini)	0.828	0.632	0.652	0.645	0.606	0.604	0.366	0.496	0.491
KATE (o3-mini, 5)	0.884	0.714	0.708	0.573	0.555	0.554	0.520	0.622	0.624

“(40)”/“(5)” = number of in-context shots; “(ft)” = fine-tuned

**Table 2:** Over- and under-triage rates (%) for the same models as in Table 1. Over-triage = predicted more urgent than ground truth; under-triage = predicted less urgent. Best values per column are **bolded**; second-best are underlined

	ESI-Handbook		KTAS		MIMIC	
Methodology	Over	Under	Over	Under	Over	Under
LogReg	N.A.	N.A.	12.1	42.5	17.7	23.7
XGBoost	N.A.	N.A.	**7.9**	57.0	17.9	22.8
BioBERT	N.A.	N.A.	16.9	34.7	13.3	18.8
Vanilla	**4.5**	16.9	35.2	12.8	11.4	31.8
AutoCoT	**4.5**	12.4	39.9	**9.5**	9.4	34.0
CoT	**4.5**	14.6	35.1	12.8	13.3	35.7
FewShot (40)	5.6	11.2	30.9	17.3	23.4	20.3
FewShotCoT (40)	16.9	12.4	23.0	20.9	22.8	18.4
KATE (40)	9.0	**9.0**	21.2	18.0	18.4	**14.3**
KATECoT (40)	12.4	11.2	20.0	21.1	21.2	14.8
Llama3.1–8B (ft)	30.1	44.0	21.7	13.3	N.A.	N.A.
Qwen3–8B (ft)	19.1	12.0	23.6	13.5	N.A.	N.A.
Vanilla (o3-mini)	9.0	25.8	21.2	18.3	**7.5**	43.4
KATE (o3-mini, 5)	12.4	16.9	26.1	18.5	19.4	18.2

**Table 3:** Counterfactual analysis under **vanilla prompting**: average acuity assigned to patients of each demographic. Lower scores mean higher priority. Per-column **bold**/underline mark the most/least prioritized demographic. “All” rows give the across-race average within each sex.

Demographic	Llama	Gemini	gpt-4o	gpt-4o	claude-3	claude-3	o3
	3.1–70B	2.0	mini		haiku	sonnet	mini
*Men*							
All	2.478	2.860	2.759	3.018	2.591	2.948	2.869
American Indian	**2.458**	2.863	2.753	3.181	2.800	3.000	2.858
Asian	2.468	2.890	2.769	3.045	2.500	3.095	2.853
Black	2.474	2.895	2.584	2.931	2.566	2.714	2.879
Hispanic	2.495	**2.811**	2.846	**2.886**	2.683	2.928	2.863
Native Hawaiian	**2.458**	2.874	2.553	3.022	2.216	2.857	2.905
White	2.516	2.832	3.046	3.045	2.783	3.095	2.858
*Women*							
All	2.486	2.865	2.653	3.064	2.436	2.682	2.865
American Indian	2.463	2.874	2.692	3.181	2.516	2.785	2.863
Asian	2.495	2.874	2.707	3.045	2.366	2.642	2.847
Black	2.479	2.858	2.584	3.022	2.333	**2.523**	2.874
Hispanic	2.500	2.868	2.800	3.022	2.500	2.547	2.890
Native Hawaiian	2.474	2.847	**2.384**	3.000	**2.166**	2.595	**2.837**
White	2.505	2.868	2.753	3.113	2.733	3.000	2.879
*Significance (Bonferroni-adjusted)*							
Sex	1.00	1.00	< 0.01**	1.00	<0.01**	<0.01**	1.00
Race	<0.01**	1.00	< 0.01**	< 0.01**	<0.01**	<0.01**	1.00
Sex × Race	<0.01**	0.39	< 0.01**	0.52	<0.01**	<0.01**	1.00

Significance rows report Bonferroni-adjusted p-values from Wilcoxon/Friedman tests (* *p* < 0.05, **p < 0.01). CoT-augmented configurations are reported separately in Table 4

**Table 4:** Counterfactual analysis under **Chain-of-Thought (CoT) prompting** for the four models that support it. Cell semantics, bolding, and significance conventions match Table 3

Demographic	gpt-4o	gpt-4o	claude-3	claude-3
	mini		haiku	sonnet
*Men*				
All	2.630	2.977	2.894	3.094
American Indian	2.666	2.983	2.912	3.132
Asian	2.612	3.000	2.947	3.066
Black	2.573	2.923	2.883	3.080
Hispanic	2.627	2.940	2.947	3.073
Native Hawaiian	2.565	3.059	2.848	3.161
White	2.736	2.957	2.830	3.051
*Women*				
All	2.629	2.963	2.810	3.068
American Indian	2.666	2.889	2.824	3.154
Asian	2.589	3.042	2.824	3.029
Black	2.627	**2.881**	2.824	**3.022**
Hispanic	2.705	2.906	2.824	3.044
Native Hawaiian	**2.511**	3.093	**2.713**	3.073
White	2.674	2.966	2.853	3.088
*Significance (Bonferroni-adjusted)*				
Sex	1.00	1.00	< 0.01**	1.00
Race	< 0.01**	< 0.01**	1.00	0.247
Sex × Race	0.028*	0.01*	0.056	0.83

**Table 5:** Evaluation of Continued Pretraining Strategies on ESI-Handbook

Method	Accuracy	QWK
*Llama-3.2-3B using full-finetuning*		
Without continued pre-training	52.70%	0.8051
10 epochs	56.76%	0.8015
20 epochs	64.87%	0.8615
*Qwen3-8B using LoRA*		
Without continued pre-training	70.81%	0.8814
10 epochs	68.90%	0.9006
30 epochs	69.38%	0.8809
30 epochs (with 6 5 paraphrased mixing)	73.68%	0.8863
30 epochs (with 3 10 paraphrased mixing)	76.56%	0.9058
60 epochs (with 6 10 paraphrased mixing)	63.64%	0.8289

**Table 6:** Deployment-feasibility ablations on ESI-Handbook: model precision (Qwen3-8B) and parameter-efficient fine-tuning (Llama-3.2-3B)

Setting	Accuracy	QWK
*Precision (Qwen3-8B)*		
4-bit	43.06%	0.7294
16-bit	68.90%	0.9006
*Fine-tuning method (Llama-3.2-3B)*		
Full fine-tuning	52.70%	0.8051
Full fine-tuning + continued pre-training	56.76%	0.8015
LoRA	50.00%	0.7808
LoRA + continued pre-training	54.05%	0.8270

**Table 7:** Evaluation of Serialization Strategies on KTAS and MIMIC

	KTAS	MIMIC
Serialization	F-1 Score	F-1 Score
Commas	0.569	0.582
Natural	0.511	0.585
Newlines	0.499	0.580
Spaces	0.540	0.582

**Table 8:** Hyperparameters used for continued pretraining and supervised fine-tuning. For rows that have two values, the latter is applied for full fine-tuning experiments in our ablation studies

Hyperparameter	Continued Pretraining	Supervised Fine-tuning
Epochs	10	25*
Learning rate	4e-5 / 1e-5	2e-4 / 2e-5
Weight decay	0.01	0.01
Scheduler	Cosine	Linear / Cosine
Warm-up	5 steps / 0.1 ratio	5 steps / 0.1 ratio
Batch size	8	8
LoRA rank (*r*)	32	32
LoRA*α*	32	32

* 25 epochs chosen after validating 10, 25, and 100

## Data Availability

All datasets used in this study are publicly available. MIMIC-IV-ED is available via PhysioNet under credentialed access [46]; the MIMIC-IV-ED-Demo subset used for the counterfactual analysis is openly available without credentialing. The Korean Triage and Acuity Scale (KTAS) dataset is publicly available as released by Moon et al. [47]. The ESI Handbook case studies are drawn from the publicly available ESI Implementation Handbook [48].

## Footnotes

1
https://www.med.upenn.edu/penndna

## Code Availability

https://github.com/jiosephlee/llms-for-triage.
